# *Dichrocephala integrifolia* (L.f.) Kuntze Leaf Aqueous Extract Improves Liver Architecture in a Model of Aflatoxin-Containing Peanut-Induced Hepatotoxicity in Wistar Rats

**DOI:** 10.1155/jt/5551651

**Published:** 2025-05-18

**Authors:** Adolphe Mbatchou, Florence Ngueguim Tsofack, Jean Hubert Donfack, Raceline Kamkumo Gounoue, Michel Arnaud Mbock, Jean Philippe Tientcheu Djientcheu, Franklin Gamo Zemo, Rodrigue Ngapout Fifen, Paul Desire Djomeni Dzeufiet, Théophile Dimo

**Affiliations:** ^1^Department of Animal Biology and Physiology, University of Yaoundé I, P.O. Box 812, Yaoundé, Cameroon; ^2^Department of Pharmaceutical Sciences, University of Dschang, P.O. Box 96, Dschang, Cameroon; ^3^Department of Biochemistry, University of Douala, P.O. Box 24157, Douala, Cameroon

**Keywords:** aflatoxin B1, cytokines hepatoprotective activities, *Dichrocephala integrifolia*, liver damage

## Abstract

Consumption of aflatoxin-contaminated food is responsible for hepatotoxicity. *Dichrocephala integrifolia* (*D. integrifolia*) is used in traditional African medicine to treat various liver diseases. This study aimed to evaluate the therapeutic effects of aqueous leaf extract of *D. integrifolia* on aflatoxin-containing peanut-induced liver damage in Wistar rats. The animals were fed the standard diet (SD) or the SD supplemented with stored and poorly preserved peanuts (50:50) containing aflatoxins for 42 days. Then, animals received the diet concomitantly with *D. integrifolia* (100, 200 mg/kg), or Silybon 100 mg/kg for 14 days. At the end of the experimental period, biochemical markers, including the lipid profile, liver function markers, and proinflammatory markers, were evaluated. A histopathological analysis of the liver was performed. Semiquantitative evaluation of aflatoxin B1 in peanuts by thin-layer chromatography was carried out, and phytochemical characterization of the extract by high-performance liquid chromatography–mass spectrometry was performed. As a result, poorly stored peanuts contain 20 μg/kg aflatoxin B1/kg of peanuts. Consumption of contaminated peanuts resulted in inflammation characterized by a significant increase (*p* < 0.001) in proinflammatory cytokines (TNF-α, INF-γ, and IL-13) and a significant increase (*p* < 0.001) in transaminase activities, GGT, ALP, and levels of total bilirubin, total cholesterol, triglycerides, LDL cholesterol, and a significant decrease (*p* < 0.001) in HDL cholesterol and albumin levels. These various abnormalities were accompanied by a significant increase (*p* < 0.001) in oxidative stress. These disturbances were confirmed by hepatic cytolysis, leukocyte infiltration, and vascular congestion. Treatment with *D. integrifolia* extract at all doses tested reversed these abnormalities. These beneficial effects of the extract could be due to *β*-amyrin formate, identified in this extract, and could therefore justify the use of this plant extract in traditional medicine to manage liver diseases.

## 1. Introduction

Liver disease is a serious illness and a major cause of death worldwide, making it a real public health problem [1]. Liver damage induced by xenobiotics or inherited disorders most commonly results in hepatic cell necrosis or hepatitis [2]. Among these various factors responsible for hepatitis, aflatoxins produced by fungi and found in cereal crops are secondary metabolites that cause severe hepatotoxicity. According to the World Health Organization (WHO), approximately 600 million people worldwide fall ill and nearly 420,000 people die each year after consuming contaminated food [3]. In Africa, poverty, food insecurity, and population growth have led to large-scale grain production with little regard for quality, let alone preservation. Consumption of food contaminated with aflatoxins is responsible for 40% of liver cancer in Africa [4]. Several aflatoxins exist, the most toxic being aflatoxin B1 (AFB1), which is carcinogenic and mutagenic [5]. Aflatoxin metabolites affect cellular metabolism by inhibiting protein and lipid synthesis and carbohydrate metabolism, thereby reducing liver function. When ingested, AFB1 is bioactivated by oxidation to a reactive form, AFB1-8, 9-epoxide, which reacts with DNA, causing damage and genetic mutation [5, 6]. Improvements in lifestyle, elimination of exposure to toxic substances (food safety), use of hepatoprotective drugs, vaccination, and, in extreme cases, liver transplantation, enable populations to combat these liver diseases. Conventional drugs used to treat liver diseases are expensive and require a long course of treatment [7]. They also cause severe side effects such as asthenia, coagulopathy, and pseudo-influenza syndrome from interferon therapy [8]. These adverse effects of conventional drugs motivate the growing public interest in the use of plants as therapeutic alternatives for liver diseases. One of the plants used in Cameroonian traditional medicine for the treatment of hepatitis is *Dichrocephala integrifolia*. Previous research on *D. integrifolia* has shown that it has anticancer, antibiotic, anti-inflammatory, antioxidant, and antihelminthic activities [9–11], anxiolytic and sedative properties [12], and hepatoprotective potential [13]. The phytochemical constituents of *D. integrifolia* include sesquiterpene-lactones, diterpene-lactones, sterols, dichrocephol, dicaffeoylquinic, flavonoids, alkaloids, saponins, and anthraquinones [14]. Six compounds have been identified in this plant: stearic acid (fatty acid), stigmasta-7,22-dien-3-ol (sterol), alpha-amyrin (triterpene), epifriedelanol (derived from frideline, triterpene), methyl stearate (methylated derivative of stearic acid, fatty acid), and tritetracontane (aliphatic compound) [15]. In our previous studies, we have shown that *D. integrifolia* leaf aqueous extract is hepatoprotective and prevents liver damage in an acute model of alcohol-induced liver injury [13, 16]. Considering the widespread contamination of AFs in food and their severe hepatotoxicity, the aim of the current study was to investigate the curative effects of *D. integrifolia* leaf aqueous extract on aflatoxin food poisoning-induced liver injury in Wistar rats.

## 2. Materials and Methods

### 2.1. Plant Material

Fresh leaves of *Dichrocephala integrifolia* (L.f.) Kuntze were collected in Mendong-Yaounde (Mfoundi Division, Center Region of Cameroon), and the botanical sample was identified at the National Herbarium of Cameroon by comparison with the voucher specimen no. 65648/HNC.

### 2.2. Chemicals and Reagents

AFB1 *Aspergillus flavus* was purchased from Sigma Chemicals, Israël, diazepam (Valium 10 mg, Roche, France), ketamine (Ketamine hypochloride 50 mg, Rotex Medica, Tritau, Germany), Silybon (Silymarin 70 mg: Micro Labs Limited, India), cytokine kits, adrenaline, sulfanilamide, phosphoric acid, hydrogen peroxide, potassium dichromate, trichloroacetic acid, and thiobarbituric acid, phosphotungstic acid; zinc sulfate, sulfuric acid were purchased from Sigma Chemicals, Germany. Petroleum, ether, chloroform, and acetone were purchased from Merck, Germany.

### 2.3. Animals

Male Wistar rats (6–8 weeks old) weighing between 160 and 180 g at the beginning of the experiment were used for this study. These animals were bred at the Animal Physiology Laboratory of the University of Yaoundé I as described in previous studies [16, 17]. The SD in our laboratory consisted of 40% maize flour, 20% wheat flour, 24% fish powder, 12% groundnut, 3.5% bone powder, and salt and vitamin complex. All procedures in the present study followed the principles for the use and care of laboratory animals of the European Community guidelines (EEC Directive 2010/63/EEC) and were approved by the National Ethical Committee of Cameroon (Ref. No. Fw-IRb00001954, 04/09/2006).

### 2.4. LCMS Analysis of *Dichrocephala integrifolia* Leaf Aqueous Extract

*Dichrocephala integrifolia* leaves were harvested, dried in the shade at room temperature, and crushed. The decoction was obtained as described by Ngueguim et al. [13]. The dry extract obtained was stored at room temperature until use. Ultra-high-performance liquid chromatography coupled with mass spectrometry (QTOF Bruker, Germany) was used to identify compounds present in the aqueous leaf extract of *Dichrocephala integrifolia* as described in our previous study [18]. Briefly, high-resolution mass spectra of the extract were obtained using a spectrometer (QTOF Bruker, Germany) equipped with a heated electrospray ionization (HESI) source. The spectrometer operates in positive mode (mass range: 100–1500, with a scan rate of 1.00 Hz) with automatic gain control to provide high accuracy mass measurements within 0.40 ppm deviation using sodium format as calibrator. The following parameters were used for the experiments: spray voltage of 4.5 kV, capillary temperature of 200°C. Nitrogen was used as the sheath gas (10 L/min). The spectrometer was attached to an Ultimate 3000 (Thermo Fisher, Germany) UHPLC system consisting of an LC pump, diode array detector (DAD) (*λ*: 190–600 nm), autosampler (injection volume 10 μL), and column oven (40°C). Separation was performed using a Synergy MAX-RP 100 A (50 × 2 mm, 2.5 μ particle size) with an H_2_O (+0.1% HCOOH) (A)/acetonitrile (+0.1% HCOOH) (B) gradient (flow rate 500 μL/min, injection volume 5 μL). The sample was analyzed using a gradient program as follows: 95% A isocratic for 1.5 min, linear gradient to 100% B over 6 min, and after 100% B isocratic for 2 min, the system returned to its initial state (90% A) within 1 min and was equilibrated for 1 min. Peak identification was performed by comparing the peaks in the samples with those in the literature data available in the SciFinder database.

### 2.5. Semiquantitative Evaluation of AFB1 in Peanut Seeds

Thin-layer chromatography (TLC) was used to estimate the amount of AFB1 in moldy groundnut seeds collected from the local market in Yaoundé. The sample preparation method described by Diakité et al. [19] was used in this experiment. Briefly, the procedure consists of four steps: extraction; deproteinization; delipidation; and detection of AFB1.

#### 2.5.1. Extraction

Market peanuts were ground into a paste. Twenty grams of the paste or peanut without aflatoxins but experimentally contaminated with pure AFB1 (20 μL/kg) were separately placed in 250-mL Erlenmeyer flasks, to which 100 mL of a methanol/water mixture (80:20) was added. After magnetic stirring for 15 min, the filtrate was obtained by filtration through Whatman No. 6 paper (filtrate 1).

#### 2.5.2. Deproteinization

A volume of 45 mL of a mixture of phosphotungstic acid (5 g) and zinc sulfate (15 g) was added to 50 mL of filtrate 1. The solution was made up to 100 mL with distilled water. The mixture was filtered through Whatman No. 6 paper to obtain filtrate 2.

#### 2.5.3. Delipidation

Lipids were removed from filtrate 2 by extraction in a separating funnel with 10 mL of petroleum ether. This process was repeated three times to obtain the aqueous phase containing aflatoxins, including AFB1.

#### 2.5.4. Aflatoxin Extraction and Detection

Aflatoxins were extracted from Extract 1 with 10 mL of chloroform in a separating funnel. This procedure was repeated three times, and the chloroform extract was collected and concentrated using a rotary evaporator (Laborota 4000 efficient type Heidolph), giving a volume of approximately 0.5 mL (Extract 2).

For the detection of AFB1in groundnut paste extracts, aluminum TLC plates (MERCK, Germany) coated with G60 silica gel stationary phase (thickness, 0.25 mm and dimensions 10 cm × 20 cm and 20 cm × 20 cm) were used. For peanut pastes contaminated with AFB1 (20 μg/kg, 10 μg/kg, 5 μg/kg, 2.5 μg/kg, and 1.25 μg/kg) and peanut pastes from the market (T1 and T2), 5 μL of each Extract 2 obtained was applied to the plate in the presence of an equal volume of AFB1 standard (25 μg/mL). The plate was eluted with a binary solvent system chloroform/acetone (90:10; v/v), and then the spots corresponding to aflatoxins were visualized in a dark room under UV radiation produced by a lamp at *λ* = 365 nm. At the end of the migration of the compounds by TLC, the frontal ratio (Rf) was calculated for each sample.

### 2.6. Evaluation of Curative Effects of Aqueous Extract of *Dichrocephala integrifolia* on Aflatoxin-Induced Hepatotoxicity

#### 2.6.1. Induction of Hepatotoxicity

Forty male Wistar rats were divided into four main groups: One group consisted of 5 rats receiving standard chow diet; The second group consisted of 5 animals receiving 50% SD and 50% peanut paste containing acetonitrile (800 μL/kg of peanuts) (SD + Can), the third group consisted of 5 animals receiving 50% SD and 50% peanut paste contaminated with AFB1 (20 μg/kg of peanuts) (SD + AFB1), and the fourth group consisted of 25 animals receiving 50% SD and 50% peanut paste (collected from the market) containing aflatoxins (SD + PCA). The animals received the different experimental diets for 42 days.

##### 2.6.1.1. Treatment

At the end of this induction period, the first three groups received distilled water (10 mL/kg) concurrently with their initial diets, while the fourth group was divided into five further groups of five rats each and treated as follows:

One group received distilled water (10 mL/kg) concomitantly with a diet containing aflatoxins (SD + PCA); one group of animals received distilled water (10 mL/kg) concomitantly with the SD (satellite control); another group received Silybon at the dose of 100 mg/kg concomitantly with a diet containing aflatoxins (SD + PCA + Silybon 100); two test groups received the aqueous leaf extract of *D. integrifolia* at the dose of 100 mg/kg (SD + PCA + Di 100) or 200 mg/kg (SD + PCA + Di 200) concomitantly with a diet containing aflatoxins.

**Figure 1:**
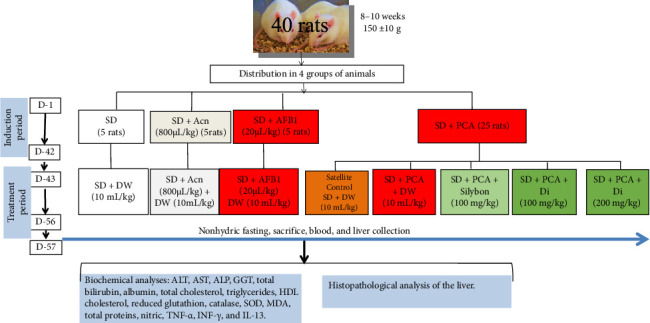
Flowchart of the experimental protocol. Standard diet (SD): animals fed a diet consisting of standard laboratory chow; SD + Can 800: animals fed standard laboratory chow with acetonitrile (800 μL/kg); SD + AFB1: animals fed standard chow with pure AFB1 (20 μg/kg); SD + PCA + DW: animals fed a diet consisting of 50% standard chow and 50% aflatoxin-containing peanut paste and given distilled water; satellite control: animals fed a normal diet with distilled water after poisoning with aflatoxin-containing peanut paste; SD + PCA + Silybon 100 mg/kg: animals fed a diet consisting of 50% standard chow and 50% aflatoxin-containing groundnut paste treated with Silybon (100 mg/kg); SD + PCA + Di 100 or SD + PCA + Di 200: animals fed a diet consisting of 50% standard chow and 50% aflatoxin-containing groundnut paste treated with the aqueous extract of the leaves of *Dichrocephala integrifolia* at a dose of 100 or 200 mg/kg.

The treatment was administered orally once daily for 14 days. Food consumption and body weight of the animals were assessed every 3 days throughout the experimental period (Figure 1). At the end of treatment, animals were anesthetized with diazepam (10 mg/kg) and ketamine (50 mg/kg) and sacrificed. Blood was collected and centrifuged at 3000 rpm for 15 min, and the serum was stored for further evaluation of biochemical parameters. Liver was also collected and weighed. A section of the liver was homogenized in cold Tris–HCl buffer (pH 7.4) at a ratio of 20% (w/v) and then centrifuged at 3000 rpm at 4°C for 25 min; the supernatant was collected in tubes and stored at −20°C for further biochemical analysis. A section of the liver was fixed in buffered formaldehyde (10%) for histopathologic analysis.

#### 2.6.2. Evaluation of Some Liver Function Markers and Lipid Profile

Serum biochemical parameters of liver function (aspartate aminotransferase (AST), alanine aminotransferase (ALT), gamma-glutamyl transferase (GGT), and alkaline phosphatase (ALP) activities; total bilirubin and albumin levels) were evaluated using commercial diagnostic kits (Labkit, Spain). In addition, a lipid profile (total cholesterol, triglycerides, and HDL cholesterol levels) was performed in the serum sample according to the manufacturing protocol of the diagnostic kits (Labkit, Spain). LDL cholesterol was determined as indicated by the diagnostic kits and the atherogenic index was determined according to the formula used by Bilanda et al. [20].

#### 2.6.3. Evaluation of Some Serum Proinflammatory Cytokine Concentrations

Some proinflammatory markers (TNF-α, INF-γ, and IL-13) were evaluated in serum samples using rat enzyme-linked immunosorbent assay (ELISA) kits according to the manufacturer's protocol. The wells were precoated with a specific capture (primary) antibody for each cytokine. Briefly, after standard reconstitution, 100 μL of standard or samples were added to each precoated well. After gently tapping the frame plate, the microplates were covered and incubated (2 h) at room temperature. After incubation, each well was gently washed 4 times with wash solution and blotted dry with clean absorbent paper. Then, 100 μL of the prepared detection antibody was added to each well. The preparation was incubated for 1 h at room temperature with gentle shaking and then washed 4 times as described above. Then, 100 μL of prepared streptavidin solution was added to each well and the preparation was incubated for 45 min at room temperature with gentle shaking and then washed 4 times as described above. And, 100 μL of TBM substrate reagent was added to each well and incubated for 30 min at room temperature in the dark with gentle shaking. Then, 50 μL of stop solution (sulfuric acid) was added to each well to stop the reaction, and the absorbance was immediately read at 450 nm using a microplate reader. Cytokine levels were expressed in pg/mL and determined from the corresponding calibration curves.

#### 2.6.4. Evaluation of Some Oxidative Stress Biomarkers

Oxidative stress biomarkers such as reduced glutathione (GSH) level, catalase activity, nitrite concentration, superoxide dismutase (SOD) level, and malondialdehyde (MDA) level were evaluated in liver tissue homogenates using the methods described by Ellman [21], Sinha [22], Fermor [23], Misra and Fridovish [24], and Wilbur [25], respectively.

#### 2.6.5. Histopathological Analysis of the Liver

The liver was fixed in 4% buffered formalin. Subsequently, the liver was subjected to different stages of dehydration and embedded in paraffin blocks. Histopathologic analysis of the liver was performed on 5-μm sections of paraffin-embedded tissue after hematoxylin–eosin staining [26]. The sample from each animal was scored using a semiquantitative scoring system for murine liver disease models technique [27, 28] based on visualization of the area with a minimum of 10 fields per animal. Hepatic changes considered were inflammation, fibrosis, and cytolysis. In addition, the severity of abnormalities was scored as follows: 0 = absence of lesions; 1 = minimal (1%–10% of tissue section affected); 2 = mild (11%–25%); 3 = moderate (26%–45%); and 4 = severe (> 45) [28].

### 2.7. Statistical Analysis

Data are expressed as mean ± S.E.M. Statistical analysis was performed using one-way ANOVA followed by Turkey post-test (GraphPad Prism, Version 8.01). The *p* value < 0.05 was considered significant.

## 3. Results

### 3.1. Semiquantitative Evaluation of AFB1 in Peanut Seeds by TLC

**Figure 2:**
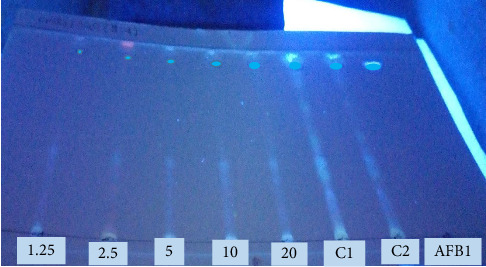
Expression of the level of contamination of the peanut paste (white matrix) by AFB1 standard. 1.25, 2.5, 5, 10, and 20: levels of contamination of peanut pastes by aflatoxin B1 in μg/kg; C1 and C2: peanut pastes from the local market; AFB1: aflatoxin B1, standard (25 μg/mL).

The analysis of the chromatograms obtained by TLC of the extracts of experimentally contaminated peanut pastes showed blue fluorescent spots with a frontal report (Fr) ∼ 0.88 for all levels of contamination (20 μg/kg, 10 μg/kg, 5 μg/kg, 2.5 μg/kg, and 1.25 μg/kg). Blue fluorescence (under UV light, 365 nm) indicates the presence of AFB1 and was comparable to pure AFB1 (standard, 25 μg/mL). The level of AFB1 contamination of the peanut paste was directly proportional to the intensity of fluorescence observed (Figure 2). Observing the fluorescence intensity of samples (1.25, 2.5, 5, 10, and 20) contaminated with AFB1 at different doses, and in comparison with peanut paste from the local market (C1 and C2), the dose of AFB1 in C1 and C2 is estimated to be approximately 20 μg/kg.

### 3.2. Effects of the Aqueous Extract of *Dichrocephala integrifolia* on Aflatoxin-Induced Hepatotoxicity

#### 3.2.1. Effects of the Aqueous Extract of *Dichrocephala integrifolia* on the Body Weight Evolution of Animals

**Figure 3:**
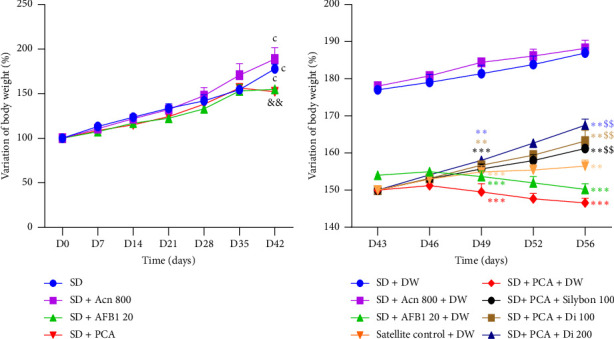
Effects of the aqueous extract of *Dichrocephala integrifolia* leaf on the variation of body weight change before (a) and after treatments (b). Each point is expressed as mean ± SEM, 5 ≥ *n* ≤ 25, ^∗∗^*p* < 0.01; ^∗∗∗^*p* < 0.001 significant difference compared to animals fed the standard diet. ^$$^*p* < 0.01 significant difference compared to animals fed the PCA diet. ^c^*p* < 0.001 significant difference compared to the baseline value during the hepatotoxicity induction phase. ^&&^*p* < 0.01 significant difference compared to the standard diet or SD + Acn groups. SD: standard diet, animals fed a diet consisting of standard laboratory chow; SD + Acn 800: animals fed a diet consisting of standard chow with acetonitrile (800 μL/kg); SD + AFB1 20: animals fed a diet consisting of standard chow with pure AFB1 (20 μg/kg); SD + PCA: animals fed a diet consisting of 50% standard chow and 50% peanut paste containing aflatoxin; satellite control + DW: animals fed a normal diet supplemented with distilled water after poisoning with aflatoxin-containing groundnut paste; SD + PCA + Silybon 100: animals fed a diet consisting of 50% standard chow and 50% aflatoxin-containing groundnut paste supplemented with Silybon (100 mg/kg); SD + PCA + Di 100 or SD + PCA + Di 200: animals fed a diet consisting of 50% standard chow and 50% aflatoxin-contaminated groundnut paste with the aqueous extract of the leaves of *Dichrocephala integrifolia* at a dose of 100 or 200 mg/kg. DW: distilled water administered at 10 mL/kg.

Figure 3 shows the variation in body weight of the animals during the induction period of hepatotoxicity (Figure 3(a)) and the effect of the plant extract (Figure 3(b)). There was a significant increase (*p* < 0.001) in body weight of the 42 days of each group compared to their initial value. During the 42-day induction of hepatotoxicity, a significant decrease (*p* < 0.01) in body weight was observed in the groups receiving AFB1- or PCA-contaminated diets compared to the SD group or the SD + Acn group. During the treatment phase, a significant reduction in body weight (*p* < 0.01-*p* < 0.001) was observed, in the SD + AFB1 and SD + PCA groups from days 49 to 56 of the experimental period compared to the SD group (Figure 3(b)). The administration of *D. integrifolia* at all the test doses, as well as Silybon (100 mg/kg), prevented (*p* > 0.05) the loss of body weight in the animal compared to the SD + PCA or SD + AFB1 groups.

#### 3.2.2. Effects of the Aqueous Extract of *Dichrocephala integrifolia* on Food Intake

**Figure 4:**
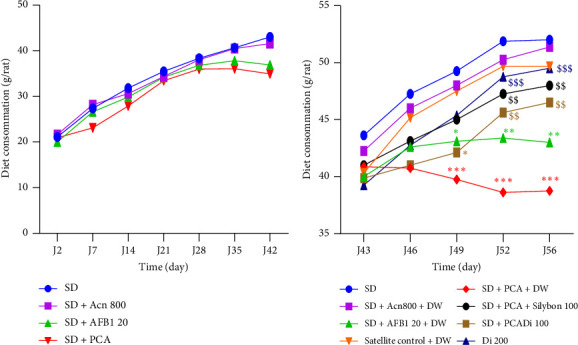
Effects of the aqueous extract of *Dichrocephala integrifolia* on food intake before (a) and after (b) treatments. Each point represents the food intake of the animals expressed as mean ± SEM, 5 ≥ *n* ≤ 25, ^∗^*p* < 0.05; ^∗∗^*p* < 0.01; ^∗∗∗^*p* < 0.001 significant difference compared to animals fed the standard diet. ^$$^*p* < 0.01; ^$$$^*p* < 0.001 significant difference compared to animals fed the PCA diet. SD: standard diet, animals fed a diet consisting of standard laboratory chow; SD + Acn 800: animals fed a diet consisting of standard chow with acetonitrile (800 μL/kg); SD + AFB1 20: animals fed a diet consisting of standard chow with pure AFB1 (20 μg/kg); SD + PCA: animals fed a diet consisting of 50% standard chow and 50% peanut paste containing aflatoxin; satellite control + DW: animals fed a normal diet supplemented with distilled water after poisoning with aflatoxin-containing groundnut paste; SD + PCA + Silybon 100: animals fed a diet consisting of 50% standard chow and 50% aflatoxin-containing groundnut paste supplemented with Silybon (100 mg/kg); SD + PCA + Di 100 or SD + PCA + Di 200: animals fed a diet consisting of 50% standard chow and 50% aflatoxin-contaminated groundnut paste with the aqueous extract of the leaves of *Dichrocephala integrifolia* at a dose of 100 or 200 mg/kg. DW: distilled water administered at 10 mL/kg.

The effect of peanut containing aflatoxin and *D. integrifolia* aqueous extract on food intake is shown in Figure 4. Consumption of peanut with aflatoxin for 42 days resulted in a reduction in food intake, although not significant (Figure 4(a)). However, compared with the SD group, consumption of aflatoxin-contaminated peanut for an additional 14 days resulted in a significant reduction in food intake of 11.56% (*p* < 0.05) and 20. 4% (*p* < 0.001) on Day 49; 12.15% (*p* < 0.01) and 23.01% (*p* < 0.001) on Day 52%; and 17.3% (*p* > 0.01) and 25.48% (*p* < 0.001) on Day 56 for the SD + AFB1 and SD + PCA groups, respectively (Figure 4(b)). Administration of the extract at all test doses, as well as Silybon (100 mg/kg), prevented the decrease in food consumption compared to the SD + PCA group (Figure 4(b)). The extract induced a significant increase in food consumption of 30.48% (*p* < 0.01) and 34.59% (*p* < 0.001) on Day 52 and 33.41% (*p* < 0.01) and 41.74% (*p* < 0.001) on Day 56 at the doses of 100 and 200 mg/kg, respectively.

#### 3.2.3. Effects of the Aqueous Extract of *Dichrocephala integrifolia* on Relative Liver Weight

**Figure 5:**
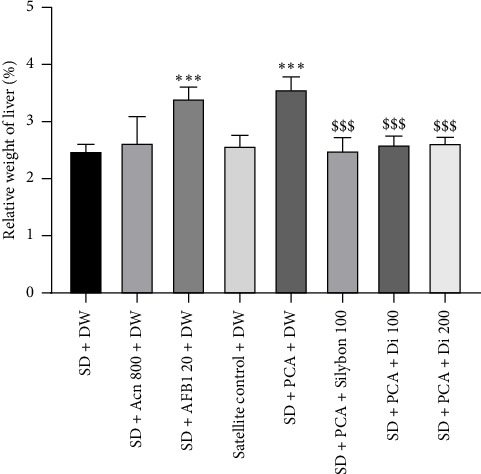
Effects of the aqueous extract of *Dichrocephala integrifolia* leaf on relative liver weight. Each bar represents the mean ± SEM, *n* = 5. ^∗∗∗^*p* < 0.001 significant difference compared to animals fed the standard diet; ^$$$^*p* < 0.001 significant difference compared to animals fed peanut-containing aflatoxin (PCA) diet. SD: standard diet, animals fed a diet consisting of standard laboratory chow; SD + Acn 800: animals fed a diet consisting of standard chow with acetonitrile (800 μL/kg); SD + AFB1 20: animals fed a diet consisting of standard chow with pure AFB1 (20 μg/kg); SD + PCA: animals fed a diet consisting of 50% standard chow and 50% peanut paste containing aflatoxin; satellite control + DW: animals fed a normal diet supplemented with distilled water after poisoning with aflatoxin-containing groundnut paste; SD + PCA + Silybon 100: animals fed a diet consisting of 50% standard chow and 50% aflatoxin-containing groundnut paste supplemented with Silybon (100 mg/kg); SD + PCA + Di 100 or SD + PCA + Di 200: animals fed a diet consisting of 50% standard chow and 50% aflatoxin-contaminated groundnut paste with the aqueous extract of the leaves of *Dichrocephala integrifolia* at a dose of 100 or 200 mg/kg. DW: distilled water administered at 10 mL/kg.

Consumption of standard chow containing AFB1 or groundnut paste containing aflatoxin for 56 days induced a significant increase (*p* < 0.001) in relative liver weight by 37.17% and 43.63%, respectively, compared to the standard chow group (Figure 5). Coadministration of the aqueous leaf extract of *D. integrifolia* (100–200 mg/kg) or Silybon (100 mg/kg) with the aflatoxin-containing peanut paste for 14 days induced a significant decrease (*p* < 0.001) in the relative weight of the liver compared to the SD + PCA group. The decrease was 26.85% and 26.19% for 100 mg/kg and 200 mg/kg, respectively, and 30.02% for Silybon.

#### 3.2.4. Effects of the Aqueous Extract of *Dichrocephala integrifolia* on Some Parameters of Liver Function

Analysis of Table 1 showed that the diet supplemented with AFB1 or PCA induced a significant increase (*p* < 0.001) in serum levels of ALT, AST, PAL, GGT, and total bilirubin and a significant decrease (*p* < 0.001) in albumin compared to the animals receiving the standard chow diet. Treatment of animals receiving SD + PCA with the aqueous leaf extract of *D. integrifolia* at the doses of 100 and 200 mg/kg, as well as Silybon 100 mg/kg, significantly reversed (*p* < 0.01-*p* < 0.001) these adverse effects induced by aflatoxin-containing peanut in animals compared to the SD + PCA group.

##### 3.2.4.1. Effects of the Aqueous Extract of *Dichrocephala integrifolia* on Some Parameters of the Lipid Profile

As shown in Table 2, consumption of aflatoxin-containing peanut or AFB1 for 56 days induced a significant increase (*p* < 0.001) in serum levels of total cholesterol, triglycerides, LDL cholesterol, and atherogenic index as compared to animals with standard chow group. Co-administration of the aqueous leaf extract of *D. integrifolia* at all the doses for 14 days with the aflatoxin-containing peanut diet induced a significant (*p* < 0.01-*p* < 0.001) decrease in serum levels of total cholesterol, triglycerides, atherogenic index, and LDL cholesterol as compared to the SD + PCA group. Daily administration of Silybon at 100 mg/kg significantly (*p* < 0.001) reduced the levels of total cholesterol, triglycerides, LDL cholesterol, and atherogenic index.

#### 3.2.5. Effects of the Aqueous Extract of *Dichrocephala integrifolia* on Some Proinflammatory Parameters

**Figure 6:**
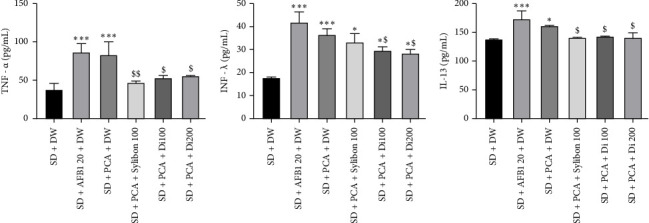
Effects of the aqueous extract of *Dichrocephala integrifolia* on serum levels of tumor necrosis factor alpha (TNF-α, (a)), interferon gamma (INF-γ, (b)) and interleukin 13 (IL-13, (c)) on aflatoxin-poisoned rats. Each bar expresses mean ± SEM, *n* = 5, ^∗^*p* < 0.05; ^∗∗^*p* < 0.01; ^∗∗∗^*p* < 0.001; significant difference compared to animals fed the standard diet. ^$^*p* < 0.05; ^$$^*p* < 0.01; significant difference compared to animals fed the PCA diet. SD: standard diet, animals fed a diet consisting of standard laboratory chow; SD + Acn 800: animals fed a diet consisting of standard chow with acetonitrile (800 μL/kg); SD + AFB1 20: animals fed a diet consisting of standard chow with pure AFB1 (20 μg/kg); SD + PCA: animals fed a diet consisting of 50% standard chow and 50% peanut paste-containing aflatoxin; satellite control + DW: animals fed a normal diet supplemented with distilled water after poisoning with aflatoxin-containing groundnut paste; SD + PCA + Silybon 100: animals fed a diet consisting of 50% standard chow and 50% aflatoxin-containing groundnut paste supplemented with Silybon (100 mg/kg); SD + PCA + Di 100 or SD + PCA + Di 200: animals fed a diet consisting of 50% standard chow and 50% aflatoxin-contaminated groundnut paste with the aqueous extract of the leaves of *Dichrocephala integrifolia* at a dose of 100 or 200 mg/kg. DW: distilled water administered at 10 mL/kg.

Consumption of aflatoxin-containing peanut or AFB1 for 56 days resulted in a significant increase (*p* < 0.001-*p* < 0.05) in serum levels of TNF-α (138.17% and 128.76%), INF-γ (108.79% and 119.65%, respectively), and for IL-13 (by 24.92% and 16.57%, respectively) compared to animals receiving SD group (Figures 6(a), 6(b), 6(c)). The extract administered at doses of 100 and 200 mg/kg for 14 days induced a significant decrease (*p* < 0.05) in serum levels of TNF-α (by 36.13% and 35.23%, respectively) (Figure 6(a)), INF-γ (by 19,78% and 22,47%, respectively) (Figure 6(b)), and IL-13 (by 11.65% and 12.44%, respectively) (Figure 6(c)) compared to animals of SD + PCA group. Silybon at a dose of 100 mg/kg, used as a reference drug, also significantly (*p* < 0.05) reduced TNF-α (*p* < 0.01) and IL-13 (*p* < 0.05) concentrations as compared to the SD + PCA group.

#### 3.2.6. Effects of the Aqueous Extract of *Dichrocephala integrifolia* on Some Oxidative Stress Parameters

**Figure 7:**
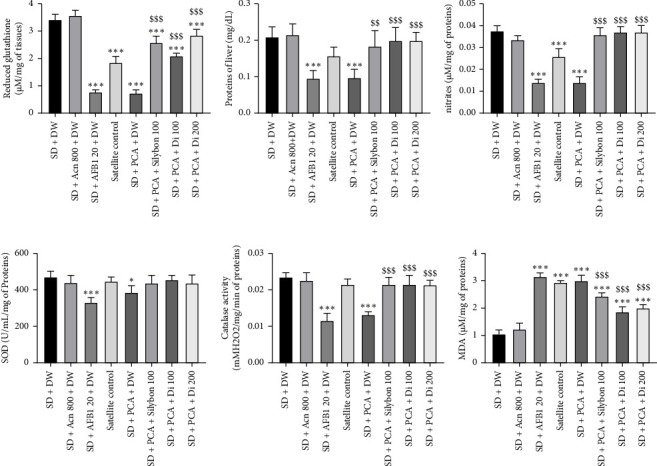
Effects of the aqueous extract of *Dichrocephala integrifolia* on reduced glutathione (a), total proteins (b), nitrite (c), SOD (d), catalase (e), and MDA (f) levels in the liver of aflatoxin-poisoned rats. Each bar represents mean ± SEM, *n* = 5, ^∗^*p* < 0.05; ^∗∗^*p* < 0.01; ^∗∗∗^*p* < 0.001; significant difference compared to animals fed the normal diet. ^$^*p* < 0.05; ^$$^*p* < 0.01; ^$$$^*p* < 0.001; significant difference compared to animals fed the PCA diet. SD: standard diet, animals fed a diet consisting of standard laboratory chow; SD + Acn 800: animals fed a diet consisting of standard chow with acetonitrile (800 μL/kg); SD + AFB1 20: animals fed a diet consisting of standard chow with pure AFB1 (20 μg/kg); SD + PCA: animals fed a diet consisting of 50% standard chow and 50% peanut paste-containing aflatoxin; satellite control + DW: animals fed a normal diet supplemented with distilled water after poisoning with aflatoxin-containing groundnut paste; SD + PCA + Silybon 100: animals fed a diet consisting of 50% standard chow and 50% aflatoxin-containing groundnut paste supplemented with Silybon (100 mg/kg); SD + PCA + Di 100 or SD + PCA + Di 200: animals fed a diet consisting of 50% standard chow and 50% aflatoxin-contaminated groundnut paste with the aqueous extract of the leaves of *Dichrocephala integrifolia* at a dose of 100 or 200 mg/kg. DW: distilled water administered at 10 mL/kg.

As shown in Figure 7, supplementation of the SD with AFB1 (SD + AFB1) or peanut containing aflatoxins induced a significant decrease (*p* < 0.05–*p* < 0.001) in hepatic levels of reduced glutathione (by 78.12% and 78.07%, respectively) (Figure 7), total proteins (by 54.99% and 53.88%, respectively) (Figure 7(b)), nitrites (by 61.94% and 62.04%, respectively) (Figure 7(c)), SOD (by 29.35% and 18.69%, respectively) (Figure 7(d)), and catalase activities (by 49.58% and 42.38%, respectively) (Figure 7(e)), whereas a significant increase (*p* < 0.001) in MDA levels (91.63% and 62.94%, respectively) (Figure 7(f)) was observed compared to the SD group. The extract at all doses as well as Silybon (100 mg/kg) significantly (*p* < 0.001) reversed all these parameters (except for SOD activity where the increase was not significant) compared to the aflatoxin-containing peanut group.

#### 3.2.7. Effects of the Aqueous Extract of *Dichrocephala integrifolia* on Histological Score and Liver Microarchitecture

Table 3 summarizes the histological score of poisoned rats treated with aqueous leaf extract of *D. integrifolia*. Inflammation, fibrosis, and cytolysis were severe in the group receiving aflatoxin-containing peanut (SD + PCA + DW) as well as in the group receiving AFB1 (SD + AFB1+DW). However, the changes were less severe in the group treated with the plant extract, with a marked effect at the dose of 200 mg/kg, despite the presence of mild inflammation.

**Figure 8:**
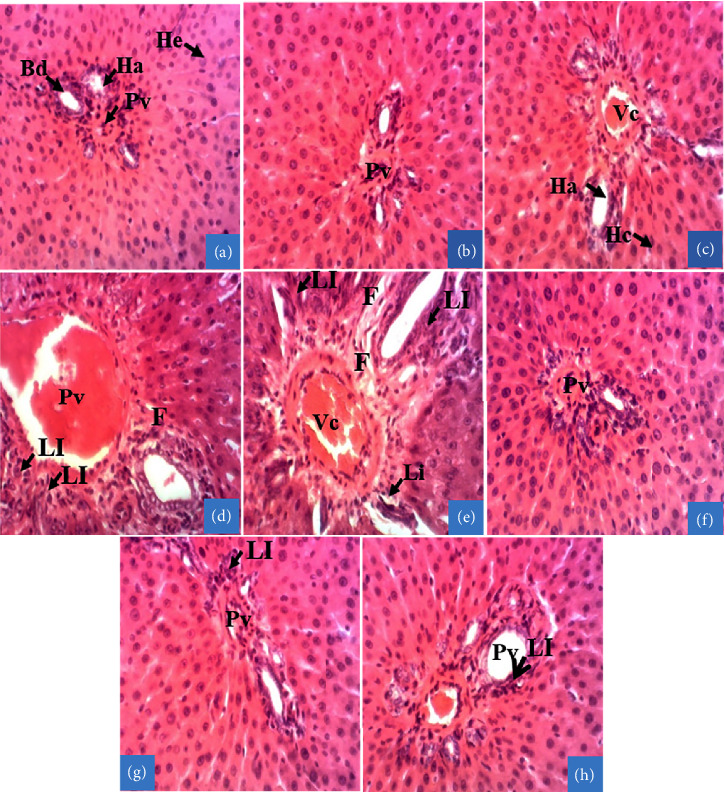
Effects of the aqueous extract of *Dichrocephala integrifolia* on liver microstructure. Photomicrographs of the liver; hematoxylin–eosin staining (×200). (a) = Standard diet (SD): animals fed a diet consisting of standard laboratory chow; (b) = SD + Acn: animals fed a diet consisting of standard chow with acetonitrile; (c) = SD + AFB1: animals fed a diet consisting of standard chow with pure AFB1 (20 μg/kg); (d) = satellite control: animals fed a normal diet with distilled water after poisoning with aflatoxin-containing peanut paste, (e) = SD + PCA: animals fed a diet consisting of 50% standard chow and 50% peanut paste-containing aflatoxin, (f) = Silybon 100 mg/kg: animals fed a diet consisting of 50% standard chow and 50% aflatoxin-containing peanut paste with Silybon (100 mg/kg); (g) = Di 100 or (h) = 200 mg/kg: animals fed a diet consisting of 50% standard chow and 50% aflatoxin-containing peanut paste with the aqueous extract of the leaf of *Dichrocephala integrifolia* at a dose of 100 or 200 mg/kg. Pv = portal vein; He = hepatocyte; Ha = hepatic artery; Bd = bile duct; Vc = vascular congestion; Li = leukocyte infiltration. F: fibrosis.

Figure 8(a) shows the liver microarchitecture of animals in the SD group, showing a normal parenchyma with a hepatic bile duct and hepatocytes separated by sinusoidal capillaries. Compared with the SD group, analysis of the liver microarchitecture of animals in the SD + AFB1 (Figure 8(c)) or SD + PCA (Figure 8(e)) groups showed that supplementation of the SD with aflatoxins induced parenchymal disorganization, vascular congestion, and leukocyte infiltration with fibrosis. The animals receiving *D. integrifolia* extract at the dose of 100 mg/kg (Figure 8(g)) or 200 mg/kg (Figure 8(h)) for 14 days showed a liver microarchitecture very close to that of a normal rat, despite the presence of vascular congestion at the dose of 100 mg/kg. The liver section from animals treated with Silybon 100 mg/kg (Figure 8(f)) also shows normal microarchitecture. However, the presence of slightly dilated sinusoidal capillaries in the liver of these animals treated with Silybon or the plant extract should be noted. This dilation is more pronounced in animals of the SD + AFB1 and SD + PCA groups than in those receiving the aqueous extract of D. integrifolia as well as Silybon 100 mg/kg.

### 3.3. Compounds Contained in the Aqueous Leaf Extract of *Dichrocephala integrifolia*

**Figure 9:**
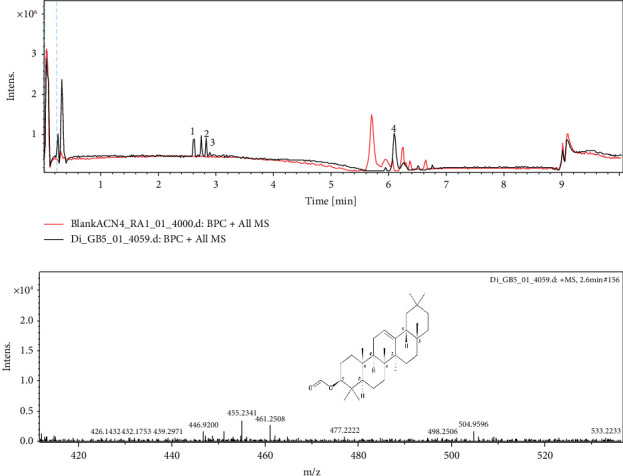
Mass spectrum of the aqueous leaf extract of *Dichrocephala integrifolia*.

Ultra-high-performance liquid chromatography coupled to mass spectrometry (UHPLC-MS) of the aqueous leaf extract of *Dichrocephala integrifolia* revealed the presence of 4 major peaks (Figure 9) with a retention time of less than 7 min. The combination of MS spectral data and literature information resulted in 4 compounds (1 identified and 3 others unknown). The peak of *β*-amyrin formate appears with a retention time of 2.6 min (compound 1, [M + H] + = 455) (Table 4). *β*-Amyrin formate was previously identified in *Dichrocephala benthamii* [21].

## 4. Discussion

The aim of the present study was to evaluate the curative effects of the aqueous leaf extract of *Dichrocephala integrifolia* on aflatoxin-containing peanut diet induced liver damage in Wistar rats. The presence of AFB1 (20 μg/kg of peanut) was confirmed by chromatography and used to induce hepatotoxicity. Animals fed the AFB1 or PCA diets for 56 days showed a reduction in body weight change (although not significant) and a significant reduction in food consumption. The reduction in food consumption could explain the reduction in body weight gain in these animals. These results are in agreement with those obtained by Galtier et al. [29], who showed that AFB1 intoxication would lead to an alteration in the weight growth of rats. It has been reported that ingestion of food containing AFB1 results in alteration of intestinal integrity, which could lead to malabsorption of food, resulting in malnutrition and reduced growth in animals [30]. In addition, Trebak et al. [31] showed that AFB1 could act on the main hypothalamic neuropeptides that regulate feeding behavior, causing appetite disturbance, resulting in anorexia and weight loss. Treatment of animals receiving AFB1 with the aqueous leaf extract of *Dichrocephala integrifolia* as well as Silybon for 14 days induced a nonsignificant increase in weight change but a significant increase in food consumption. These results suggest that by increasing appetite and/or improving intestinal absorption, the extract may be able to reduce the harmful effects of aflatoxins in food.

In this study, ingestion of aflatoxin-containing foods leading to liver problems induced a significant increase in the serum activities of ALT, AST, GGT, and ALP and the level of total bilirubin, as well as a significant decrease in the level of albumin. It is known that the level of transaminases is an indicator of liver function. During intoxication with aflatoxins, the compound is metabolized to AFB1-8,9-endo-epoxide and AFB1-8,9-exoepoxide. Aflatoxin B1-8,9-exoepoxide predominates, is highly reactive, and is responsible for the toxicity of AFB1 [5, 29], which causes hepatocyte lysis characterized by an increase in ALT and AST activities [32, 33]. The increase in ALP and GGT activities and total bilirubin levels observed in aflatoxin-poisoned animals is a sign of biliary damage observed during hepatitis [33]. In fact, ALP and GGT activities can increase specifically when the bile ducts are partially or completely obstructed, preventing bile from flowing into the intestine. This results in a reduction of bile acids in the intestinal lumen and an increase in their concentration in the bloodstream. During disease, hepatocytes normally stop excreting bilirubin, causing it to accumulate in these cells. Once the liver cells are damaged, bilirubin flows into the blood [34], explaining the hyperbilirubinemia observed in animals poisoned by aflatoxins. Albumin is the major blood protein synthesized by the liver. A decrease in this parameter may be caused by malnutrition and liver and inflammatory diseases. The alteration in albumin synthesis due to hepatocyte damage could be demonstrated by the decrease in serum albumin levels in poisoned animals. Thus, the improvement in serum activities of ALT, AST, GGT, and ALP, albumin, and total bilirubin concentrations in the plant extract treatment groups shows the ability of aqueous extract of D. integrifolia to counteract the deleterious effect of aflatoxin in peanut paste in liver cells [35]. This could be justified by its anti-inflammatory activity provided by the *β*-amyrin formate present in the extract. All these adverse effects induced by aflatoxin were significantly reversed by administration of aqueous extract of D. integrifolia at all doses. It has been well demonstrated that silybin, composed mainly of silymarin, acts directly on hepatocytes, inhibiting the absorption of toxins and promoting their elimination [36]. AFB1 has been reported to cause dysregulation of lipid profile through altered gene expression and lipid disruption [36]. This is the case of the aryl hydrocarbon receptor gene, which regulates fatty acid and cholesterol biosynthesis in the liver. In fact, AFB1 suppresses the expression of the aryl hydrocarbon receptor gene, resulting in the elevation of cholesterol observed in this study; hepatic lipase, encoded by the LIPC gene, is also suppressed by AFB1, contributing to hyperlipidemia [37]. The consequence of this alteration is the development of cardiovascular diseases [37, 38]. This is consistent with our findings that aflatoxin-containing peanut induced hypertriglyceridemia and hypercholesterolemia with high atherogenicity. The aqueous extract of *D. integrifolia* at all doses, as well as Silybon, improved lipid levels close to those of animals on a SD. Thanks to its hypolipidemic properties controlled by alkaloids [39], the plant extract could correct lipid metabolism disorders caused by aflatoxin. Aflatoxin-induced hepatotoxicity results from the alteration of mitochondrial respiratory function by modifying DNA and the mitochondrial respiratory chain, generating ROS and intense oxidative stress [40, 41]. In this study, aflatoxin ingestion induced a significant decrease in catalase and SOD activities. This may be due to AFB1-induced changes in the dynamic permeability of cell membranes (lipid peroxidation), followed by release of these intracellular enzymes into the general circulation [42]. This lipid peroxidation was confirmed in this study by an increase in MDA levels in the aflatoxin-containing peanut group. Glutathione is the most abundant endogenous nonenzymatic antioxidant and a direct scavenger of reactive oxygen species. The significant decrease in reduced glutathione levels observed in the liver of aflatoxin-poisoned animals could probably be related to the oxidative stress induced by AFB1 [43]; the administration of the extract improved these parameters, suggesting its antioxidant activities. These properties of the extract could be attributed to the flavonoids and alkaloids present in the extract, which have the ability to scavenge free radicals and regenerate liver cells [33]. Nitrite levels were also significantly reduced in rats poisoned with aflatoxins. Nitrite is a derivative of nitric oxide (NO), and NO is a key signaling molecule in the cardiovascular system due to its vasodilatory properties. A decrease in nitric oxide or NO can facilitate vasoconstriction and consequently hypertension. Regarding the role of nitrites in the regulation of the cardiovascular system, a decrease in nitrite concentration could lead to portal hypertension, which is characterized by an increase in intrahepatic vascular resistance, the appearance of portal venous collateral circulation, and a hyperkinetic syndrome [44]. Administration of aqueous extract of *D. integrifolia* induced a significant increase in catalase and SOD activities, reduced glutathione and nitrite levels, and a significant reduction in MDA levels. These results suggested that the extract could protect the liver from free radicals, fight lipid peroxidation, and regulate membrane permeability. This capacity of the extract could be due to the *β*-amyrin formate identified in this extract plant by UHPLC-MS essay. It has been reported that *β*-amyrin formate is endowed with antioxidant properties that justify the various beneficial effects observed [45].

In our study, aflatoxin-poisoned animals also showed a significant increase in serum levels of TNF-α, INF-γ, and IL-13 compared to animals receiving standard chow, reflecting inflammation. AFB1 has been reported to cause inflammatory responses by inducing an increase in cytokine expression [46–48]. Indeed, AFB1-8,9-exoepoxide is able to bind to hepatic proteins, leading to inflammatory responses in which the release of TNF-α by macrophages, dendritic cells, and mast cells stimulates the expression of adhesion molecules and the production of chemokines by endothelial cells, allowing the recruitment of leukocytes to the site of inflammation. INF-γ and interleukin 13 can also stimulate the phagocytic activity of macrophages and the synthesis of antibodies, thus increasing proinflammatory cytokines [49]. These results are confirmed by histological analysis showing leukocyte infiltration, hepatic cytolysis, and vascular congestion on the liver microarchitecture of animals poisoned with aflatoxins. The aqueous extract of *D. integrifolia*, as well as Silybon, induced a significant decrease in serum levels of TNF-alpha, INF-γ, and IL-13 compared to animals fed the PCA diet. These results suggest that the extract may have anti-inflammatory properties. The *β*-amyrin formate present in the plant, which is able to inhibit the production of proinflammatory cytokines [43], may be the compound responsible for these beneficial effects of the extract. The hepatoprotective activities of the aqueous extract of *D. integrifolia* were confirmed by the analysis of the microarchitecture of the liver, which showed an architecture similar to that of animals receiving the SD.

## 5. Conclusion

In conclusion, the aqueous extract of *Dichrocephala integrifolia* protected rats exposed to aflatoxin from liver damage. The extract improved food consumption and growth of the animals. It also reduced oxidative stress and restored liver function and cytokine levels. The beneficial effects of *D. integrifolia* may be due to the antioxidant and anti-inflammatory activities of *β*-amyrin formate identified in this plant extract. These results may justify the use of this plant extract in traditional medicine for the treatment of liver diseases. Further studies are needed to clarify the exact mechanism.

## Figures and Tables

**Table 1:** Effects of the aqueous extract of *Dichrocephala integrifolia* on some serum parameters of liver function.

Parameters	Groups							
	SD	SD + Acn 800 + DW	SD + PCA + AFB1 20	Satellite control + DW	SD + PCA + DW	SD + PCA + Silybon100	SD + PCA Di 100	SD + PCA Di 200
ALT (UI/L)	61.49 ± 3.04	51.76 ± 3.21	150.84 ± 7.93^∗∗∗^	82.22 ± 3.00	177.74 ± 8.97^∗∗∗^	64.75 ± 4.15^$$$^	62.97 ± 5.07^$$$^	63.99 ± 4.59^$$$^
AST (UI/L)	79.38 ± 3.76	90.27 ± 3.01	167.98 ± 8.64^∗∗∗^	93.44 ± 6.83	178.66 ± 7.08^∗∗∗^	97.37 ± 5.57^$$$^	89.67 ± 7.93^$$$^	86.06 ± 5.57^$$$^
ALP (UI/L)	147.86 ± 6.73	160.09 ± 5.9	232.91 ± 5.18^∗∗∗^	210.38 ± 10.75^∗∗∗^	245.27 ± 11.32^∗∗∗^	171.53 ± 9.41^$$$^	182.40 ± 7.46^$$$^	159.84 ± 11.45^$$$^
GGT (UI/L)	1.53 ± 0.12	1.55 ± 0.15	5.62 ± 0.28^∗∗∗^	3.07 ± 0.22^∗∗∗^	5.56 ± 0.27^∗∗∗^	2.36 ± 0.23^$$$^	3.45 ± 0.17^∗$$$^	2.21 ± 0.19^$$$^
T. Bil (mg/dL)	0.29 ± 0.02	0.33 ± 0.01	0.55 ± 0.02^∗∗∗^	0.37 ± 0.01^∗∗∗^	0.58 ± 0.01^∗∗∗^	0.35 ± 0.01^$$$^	0.39 ± 0.02^∗∗$$$^	0.26 ± 0.01^$$$^
Albumin (mg/dL)	16.03 ± 1.08	15.4 ± 0.79	9.04 ± 0.56^∗∗∗^	12.63 ± 0.47^∗^	7.16 ± 0.35^∗∗∗^	12.93 ± 0.28^∗$$$^	11.37 ± 0.81^∗∗∗$$^	14.69 ± 0.56^$$$^

*Note:* Each value represents the mean ± SEM, *n* = 5. SD: standard diet, animals fed a diet consisting of standard laboratory chow; SD + Acn 800: animals fed a diet consisting of standard chow with acetonitrile (800 μL/kg); SD + AFB1 20: animals fed a diet consisting of standard chow with pure AFB1 (20 μg/kg); SD + PCA: animals fed a diet consisting of 50% standard chow and 50% peanut paste-containing aflatoxin; satellite control + DW: animals fed a normal diet supplemented with distilled water after poisoning with aflatoxin-containing groundnut paste; SD + PCA + Silybon 100: animals fed a diet consisting of 50% standard chow and 50% aflatoxin-containing groundnut paste supplemented with Silybon (100 mg/kg); SD + PCA + Di 100 or SD + PCA + Di 200: animals fed a diet consisting of 50% standard chow and 50% aflatoxin-contaminated groundnut paste with the aqueous extract of the leaves of *Dichrocephala integrifolia* at a dose of 100 or 200 mg/kg. DW: distilled water administered at 10 mL/kg. T. Bilirubin: total bilirubin; AST: aspartate aminotransferase; ALT: alanine aminotransferase; APL: alkaline phosphatase.

Abbreviation: GGT = gamma-glutamyl transferase.

^∗^ *p* < 0.05.

^∗∗^ *p* < 0.01.

^∗∗∗^ *p* < 0.001 significant difference compared to animals fed the standard diet.

^$$^ *p* < 0.01.

^$$$^ *p* < 0.001 significant difference compared to animals fed the PCA diet.

**Table 2:** Effects of the aqueous extract of *Dichrocephala integrifolia* on some parameters of the lipid profile.

Parameters	Groups							
	SD + DW	SD + Acn 800 + DW	SD + AFB1 20 + DW	Satellite control	SD + PCA + DW	SD + PCA + Silybon 100	SD + PCA + Di 100	SD + PCA + Di 200
Total cho (mg/dL)	39.74 ± 2.1	45.74 ± 2.05	61.87 ± 1.88^∗∗∗^	48.3 ± 1.9^∗∗∗^	62.19 ± 1.2^∗∗∗^	43.12 ± 1.51^$$$^	44.33 ± 2.93^$$$^	48.93 ± 2.48^$$^
Triglycerides (mg/dL)	34.63 ± 4.16	39.76 ± 1.59	85.06 ± 3.63^∗∗∗^	47.8 ± 2.85	83.1 ± 2.07^∗∗∗^	36.65 ± 2.56^$$$^	34.15 ± 3.63^$$$^	36.19 ± 1.77^$$$^
HDL cho (mg/dL)	14.58 ± 0.8	15.3 ± 0.96	9.47 ± 0.72	10.59 ± 1.1	8.33 ± 0.88^∗∗^	12.48 ± 1.22	12.72 ± 1.05	11.55 ± 1.19
LDL cho (mg/dL)	18.23 ± 2.39	22.49 ± 1.7	35.39 ± 2.14^∗∗∗^	28.17 ± 2.6^∗^	37.22 ± 1.6^∗∗∗^	23.31 ± 2.02^$$$^	28.77 ± 0.97^∗^	26.14 ± 2.13^$$^
Atherogenic index	2.76 ± 0.2	3.03 ± 0.2	7.11 ± 0.38^∗∗∗^	4.38 ± 0.38	7.22 ± 0.6^∗∗∗^	3.58 ± 0.33^$$$^	3.58 ± 0.37^$$$^	4.32 ± 0.24^$$$^

*Note:* Each value represents the mean ± SEM, *n* = 5. SD: standard diet, animals fed a diet consisting of standard laboratory chow; SD + Acn 800: animals fed a diet consisting of standard chow with acetonitrile (800 μL/kg); SD + AFB1 20: animals fed a diet consisting of standard chow with pure AFB1 (20 μg/kg); SD + PCA: animals fed a diet consisting of 50% standard chow and 50% peanut paste-containing aflatoxin; satellite control + DW: Animals fed a normal diet supplemented with distilled water after poisoning with aflatoxin-containing groundnut paste; SD + PCA + Silybon 100: animals fed a diet consisting of 50% standard chow and 50% aflatoxin-containing groundnut paste supplemented with Silybon (100 mg/kg); SD + PCA + Di 100 or SD + PCA + Di 200: animals fed a diet consisting of 50% standard chow and 50% aflatoxin-contaminated groundnut paste with the aqueous extract of the leaves of *Dichrocephala integrifolia* at a dose of 100 or 200 mg/kg; DW: distilled water administered at 10 mL/kg.

^∗^ *p* < 0.05.

^∗∗^ *p* < 0.01.

^∗∗∗^ *p* < 0.001 significant difference compared to animals fed the standard diet.

^$$^ *p* < 0.01.

^$$$^ *p* < 0.001 significant difference compared to animals fed the PCA diet.

**Table 3:** Histopathological score of rats fed with peanut-containing aflatoxin B1.

Groups	Parameters			
	Periportal inflammation	Portal fibrosis	Cytolysis	Score
SD	Absent	Absent	Absent	0
SD + Acn 800 + DW	Absent	Absent	Absent	0
SD + AFB1 20 + DW	Immune cell foci (50%)	Severe (50%)	Severe (50%)	4
Satellite control (SD + DW)	Diffuse immune cell infiltrate (67%)	Moderate (33%)	Absent	3
SD + PCA + DW	Diffuse immune cell infiltrate (83%)	Severe (50%)	Mild (17%)	4
SD + PCA + syl 100	Scattered immune cells (33%)	Absent	Moderate (33%)	3
SD + PCA + Di 100	Moderate (33%)	Moderate (33%)	Absent	3
SD + PCA + Di 200	Mild (20%)	Absent	Absent	2

*Note:* SD + Acn 800: animals fed a diet consisting of standard chow with acetonitrile (800 μL/kg); SD + AFB1 20: animals fed a diet consisting of standard chow with pure AFB1 (20 μg/kg); SD + PCA: animals fed a diet consisting of 50% standard chow and 50% peanut paste-containing aflatoxin; satellite control + DW: animals fed a normal diet supplemented with distilled water after poisoning with aflatoxin-containing groundnut paste; SD + PCA + Silybon 100: animals fed a diet consisting of 50% standard chow and 50% aflatoxin-containing groundnut paste supplemented with Silybon (100 mg/kg); SD + PCA + Di 100 or SD + PCA + Di 200: animals fed a diet consisting of 50% standard chow and 50% aflatoxin-contaminated groundnut paste with the aqueous extract of the leaves of *Dichrocephala integrifolia* at a dose of 100 or 200 mg/kg. DW: distilled water administered at 10 mL/kg.

**Table 4:** Compounds detected in the aqueous leaf extract of *Dichrocephala integrifolia*.

No	Time (min)	[M + H]^+^		Molecular formula	Name of compounds
		Experimental	Calculate		
1	2.6	455.2341	455.2369	C_22_ H_24_ O_6_	β-Amyrin formate
2	2.8	414.3033	414.2995	C_30_ H_39_ O	Not identified
3	2.9	481.3172	481.3160	C_27_ H_45_ O_7_	Not identified
4	6.1	447.3225	447.3258	C_31_ H_43_ O_2_	Not identified

## Data Availability

The data used to support the findings of this study are available from the corresponding author upon request.

## References

[1] Melaram R. (2021). Environmental Risk Factors Implicated in Liver Disease: A Mini-Review. *Frontiers in Public Health*.

[2] Daré B. L., Ferron J. P., Gicquel T. (2021). Once Upcon a Time Hepatotoxicity. *University of Renne, Institut of Nutrition, of Metabolism and of Cancer*.

[3] Who (2021). *Hepatitis Can’t Wait Any Longer*.

[4] Dieme E., Fall R., Sarr I., Sarr F., Traore D., Seydi M. (2017). Contamination des Céréales par L’aflatoxine en Afrique: Revue des Méthodes de Lutte Existantes. *International Journal of Brain and Cognitive Sciences*.

[5] Mona Hassan S., Sharif Mughal S., Khurram Hassan S. (2020). Cellular Interactions, Metabolism, Assessment and Control of Aflatoxins: An Update. *Computational Biology and Bioinformatics*.

[6] Castell J. V., Teresa Donato M., Gómez-Lechón M. J. (2005). Metabolism and Bioactivation of Toxicants in the Lung. The In Vitro Cellular Approach. *Experimental & Toxicologic Pathology*.

[7] WHO (2013). *WHO Urges Governments to Act against Hepatitis. World Hepatitis Day*.

[8] Parmar S. R., Vashrambhai P. H., Kalia (2010). Hepatoprotective Activity of Some Plants Extract against Paracetamol Induced Hepatotoxicity in Rats. *Journal of Herbal Medicine and Toxicology*.

[9] Mothana R. A., Gruenert R., Bednarski P. J., Lindequist U. (2009). Evaluation of the In Vitro Anticancer, Antimicrobial and Antioxidant Activities of Some Yemeni Plants Used in Folk Medicine. *Pharmazie*.

[10] Wabo P. J., Payne V. K., Mbogning T. G. (2013). *In Vitro* Anthelminthic Efficacy of *Dichrocephala integrifolia* (Asteraceae) Extracts on the Gastro-Intestinal Nematode Parasite of Mice: *Heligmosomoides Bakeri* (Nematoda, Heligmosomatidae). *Asian Pacific Journal of Tropical Biomedicine*.

[11] Fankem G. O., Fokam Tagne M. A., Noubissi P. A. (2019). Antioxydant Activity of Dichloromethane Fraction of *Dichrocephala integrifolia* in Salmonella Typhi-Infected Rats. *Journal of Integrative Medicine*.

[12] Ketcha Wanda G. J. M., Djiogue S., Gamo F. Z., Ngitedem S. G., Njamen D. (2015). Anxiolytic and Sedative Activities of Aqueous Leaf Extract of *Dichrocephala integrifolia* (Asteraceae) in Mice. *Journal of Ethnopharmacology*.

[13] Ngueguim T. F., Mbatchou A., Donfack J. H. (2016). *Dichrocephala integrifolia* (Linn.f.) O. Kuntze (Asteraceae) Leaf Aqueous Extract Prevents Ethanol-Induced Liver Damage in Rats. *Journal of Pharmacologia*.

[14] Qin F., Yan H. M., Qing X. (2015). Chemical Constituents of *Dichrocephala integrifolia*. *Chemistry of Natural Compounds*.

[15] Zhu S. H., Zhang Q. J., Chen Q., Zhou T., Yao R. J. (2010). Study on the Chemical Constituents of *Dichrocephala integrifolia*. *Zhong Yao Cai*.

[16] Adolphe M., Florence N. T., Hubert D. J., Raceline G. K., Rodrigue F. N., Theophile D. (2022). Protective Potential of Aqueous Extract From *Dichrocephala integrifolia* (Linn. f.) O. Kuntze (Asteraceae) on Blood and Biochemical Constituents in Ethanol-Induced Hepato-Nephrotoxicity in Rats. *Journal of Medicinal Plants Studies*.

[17] Bilanda D. C., Tcheutchoua Y. C., Djomeni Dzeufiet P. D. (2019). Antihypertensive Activity of *Leersia hexandra* Sw. (Poaceae) Aqueous Extract on Ethanol-Induced Hypertension in Wistar Rat. *Evidence-Based Complementary and Alternative Medicine*.

[18] Tientcheu J. P. D., Ngueguim F. T., Gounoue R. K. (2023). The Extract of *Sclerocarya birrea, Nauclea latifolia*, and *Piper longum* Mixture Ameliorates Diabetes-Associated Cognitive Dysfunction. *Metabolic Brain Disease*.

[19] Diakité A., Gouli M., N’dri D., Yapa J. (2017). Determination of Contamination by Aflatoxin B1 of Peanut Paste Consumed by Populations in Ivory Coast: Benefit of Thin Layer Chromatography. *International Journal of Brain and Cognitive Sciences*.

[20] Bilanda D. C., Dzeufiet P. D. D., Kouakep L. (2017). Bidens Pilosa Ethylene Acetate Extract Can Protect against L-NAME-Induced Hypertension on Rats. *BMC Complementary and Alternative Medicine*.

[21] Ellman G. L. (1959). Tissue Sulfhydryl Groups. *Archives of Biochemistry and Biophysics*.

[22] Sinha A. K. (1972). Colorimetric Assay of Catalase. *Analytical Biochemistry*.

[23] Fermor B., Weinberg J. B., Pisetsky D. S., Misukonis M. A., Banes A. J., Guilak F. (2001). The Effects of Static and Intermittent Compression on Nitric Oxide Production in Articular Cartilage Explants. *Journal of Orthopaedic Research*.

[24] Misra F. (1972). *Determination of the Level of Superoxide Dismutase in Whole Blood*.

[25] Wilbur K. M., Bernheim F., Shapiro O. W. (1949). Determination of Lipid Peroxidation. *Archives of Biochemistry*.

[26] Gounoue Kamkumo R., Tsakem Nangap J., Tchokouaha Yamthe L. (2020). Antimalarial Activity of the Aqueous Extract of *Euphorbia cordifolia* Elliot in Plasmodium Berghei-Infected Mice. *Asian Pacific Journal of Tropical Medicine*.

[27] Gibson-Corle K. N., Olivier A. K., Meyerholz D. K. (2013). Principles for Valid Histopathologic Scoring in Research Veterinary. *Pathology*.

[28] Fatma A. Z. A., Abdel-Maksoud F. M., Hekmat O. A. E., Ashraf Al-B., Ehab K. E. (2021). Descriptive Histopathological and Ultrastructural Study of Hepatocellular Alterations Induced by Aflatoxin B1 in Rats. *Animals*.

[29] Galtier P., More J., Bodin G. (1974). Toxins of Aspergillus ochraceus Wilhelm III. Acute Toxicity of Ochratoxin A in Rats and Adult Mice. *Annales de Recherches Veterinaires*.

[30] Yu J. (2012). Current Understanding on Aflatoxin Biosynthesis and Future Perspective in Reducing Aflatoxin Contamination. *Toxins*.

[31] Trebak F., Alaoui A., Alexandre D. (2015). Impact of Aflatoxin B1 on Hypothalamic Neuropeptides Regulating Feeding Behavior. *NeuroToxicology*.

[32] Noureddine B., Dagmar K. (2011). *Data Sheets; Liver Enzymes*.

[33] Jha A., Krithika R., Manjeet D., Verma R. J. (2013). Protective Effect of Black Tea Infusion on Aflatoxin-Induced Hepatotoxicity in Mice. *Journal of Clinical and Experimental Hepatology*.

[34] Nguepi I. S., Ngueguim T., Gounoue R., Mbatchou A., Dimo T. (2021). Curative Effects of the Aqueous Extract of *Tithonia diversifolia* (Hemsl.) A. Gray (Asteraceae) against Ethanol Induced-Hepatotoxicity in Rats. *Journal of Basic and Clinical Physiology and Pharmacology*.

[35] Vitor C., Figueiredo C. P., Hara D. B., Bento A. F., Mazzuco T. L., Calixto J. B. (2009). Therapeutic Action and Underlying Mechanisms of a Combination of Two Pentacyclic Triterpenes, α‐and β‐Amyrin, in a Mouse Model of Colitis. *British Journal of Pharmacology*.

[36] Dubé P. A., Lefebre L., Blais R. (2010). The Silymarin in Amatoxin Poisoning. *Toxicological information bulletin*.

[37] Rotimi O. A., Rotimi S. O., Duru C. U. (2017). Acute Aflatoxin B1-Induced Hepatotoxicity Alters Gene Expression and Disrupts Lipid and Lipoprotein Metabolism in Rats. *Toxicology Reports*.

[38] Hammoudeh N., Soukkarieh C., Murphy D. J., Hanano A. (2020). Involvement of Hepatic Lipid Droplets and Their Associated Proteins in the Detoxification of Aflatoxin B_1_ in Aflatoxin-Resistance BALB/C Mouse. *Toxicology Reports*.

[39] Baliga M. S., Jagentia G. C., Ullor J. N. (2002). Safety of Hydroalcoholic Extract of Sapthaparn (*Alstonia scholaris*) in Mice and Rats. *Toxicology*.

[40] Li C., Liu X., Wu J., Ji X., Xu Q. (2022). Research Progress in Toxicological Effects and Mechanism of Aflatoxin B1 Toxin. *PeerJ*.

[41] Hua Z., Liu R., Chen Y. (2021). Contamination of Aflatoxins Induces Severe Hepatotoxicity through Multiple Mechanisms. *Frontiers in Pharmacology*.

[42] Devi P. U., Ganasoundari A. (1999). Modulation of Glutathione and Antioxidant Enzymes by *Ocimum sactumand* its Role in Protection against Radiation Injury. *Indian Journal of Experimental Biology*.

[43] Ighodaro O. M., Omole J. O., Uwaifo A. O. (2010). Effects of Chronic Ethanol Administration on Body Weight, Reduced Glutathione (GSH), Malondialdehyde (MDA) Levels and Glutathione-S-Transferase Activity (GST) in Rats. *New York Science Journal*.

[44] Omar S. A., Webb A. J. (2014). Nitrite Reduction and Cardiovascular Protection. *Journal of Molecular and Cellular Cardiology*.

[45] Díaz-Ruiz G., Hernández-Vázquez L., Luna H., Wacher-Rodarte M., Navarro-Ocaña A., Navarro-Ocaña A. (2012). Growth Inhibition of *Streptococcus* From the Oral Cavity by α-Amyrin Esters. *Molecules*.

[46] Qian G., Tang L., Guo X. (2014). Aflatoxin B1 Modulates the Expression of Phenotypic Markers and Cytokines by Splenic Lymphocytes of Male F344 Rats. *Journal of Applied Toxicology*.

[47] Li Y., Ma Q. G., Zhao L. H. (2014). Effects of Lipoic Acid on Immune Function, the Antioxidant Defense System and Inflammation-Related Gene Expression of Broiler Chickens Fed Aflatoxin-Contaminated Diets. *International Journal of Molecules Sciences*.

[48] Jiang D., Liang J., Fan J. (2005). Regulation of Lung Injury and Repair by Toll-Like Receptors and Hyaluronan. *Nature Medicine*.

[49] Long M., Zhang Y., Li P. (2016). Intervention of Grape Seed Proanthocyanidin Extract on the Subchronic Immune Injury in Mice Induced by Aflatoxin B1. *International Journal of Molecular Sciences*.

